# A C-Type Lectin from *Bothrops jararacussu* Venom Disrupts Staphylococcal Biofilms

**DOI:** 10.1371/journal.pone.0120514

**Published:** 2015-03-26

**Authors:** Raphael Contelli Klein, Mary Hellen Fabres-Klein, Leandro Licursi de Oliveira, Renato Neves Feio, François Malouin, Andréa de Oliveira Barros Ribon

**Affiliations:** 1 Laboratório de Biotecnologia Molecular, Departmento de Bioquímica e Biologia Molecular, Universidade Federal de Viçosa, 36570.000, Viçosa, Minas Gerais, Brazil; 2 Laboratório de Glicobiologia, Departamento de Biologia Geral, Universidade Federal de Viçosa, 36570.000, Viçosa, Minas Gerais, Brazil; 3 Museu de Zoologia João Moojen, Departamento de Biologia Animal, Universidade Federal de Viçosa, 36570.000, Viçosa, Minas Gerais, Brazil; 4 Centre d’Étude et de Valorisation de la Diversité Microbienne (CEVDM), Département de Biologie, Faculté des Sciences, Université de Sherbrooke, Sherbrooke, Quebec, Canada; Instituto Butantan, BRAZIL

## Abstract

Bovine mastitis is a major threat to animal health and the dairy industry. *Staphylococcus aureus* is a contagious pathogen that is usually associated with persistent intramammary infections, and biofilm formation is a relevant aspect of the outcome of these infections. Several biological activities have been described for snake venoms, which led us to screen secretions of *Bothrops jararacussu* for antibiofilm activity against *S*. *aureus* NRS155. Crude venom was fractionated by size-exclusion chromatography, and the fractions were tested against *S*. *aureus*. Biofilm growth, but not bacterial growth, was affected by several fractions. Two fractions (15 and 16) showed the best activities and were also assayed against *S*. *epidermidis* NRS101. Fraction 15 was identified by TripleTOF mass spectrometry as a galactose-binding C-type lectin with a molecular weight of 15 kDa. The lectin was purified from the crude venom by D-galactose affinity chromatography, and only one peak was observed. This pure lectin was able to inhibit 75% and 80% of *S*. *aureus* and *S*. *epidermidis* biofilms, respectively, without affecting bacterial cell viability. The lectin also exhibited a dose-dependent inhibitory effect on both bacterial biofilms. The antibiofilm activity was confirmed using scanning electron microscopy. A pre-formed *S*. *epidermidis* biofilm was significantly disrupted by the C-type lectin in a time-dependent manner. Additionally, the lectin demonstrated the ability to inhibit biofilm formation by several mastitis pathogens, including different field strains of *S*. *aureus*, *S*. *hyicus*, *S*. *chromogenes*, *Streptococcus agalactiae*, and *Escherichia coli*. These findings reveal a new activity for C-type lectins. Studies are underway to evaluate the biological activity of these lectins in a mouse mastitis model.

## Introduction

A bacterial biofilm is defined as a complex and structured community of organisms enclosed in a self-produced polymeric matrix that contains exopolysaccharides, proteins, teichoic acids, enzymes, and extracellular DNA [1, 2]. Biofilms provide a firm attachment to different surfaces [3] and also provide physical protection against diverse environmental conditions, such as UV exposure, dehydration, salinity, the host immunological system, and antimicrobial agents [4, 5].

Previous studies have demonstrated that bacteria inside biofilms can be up to 1000 times more resistant to antibiotics than free-living bacteria [6, 7]. Taken together, these features make infections caused by biofilm-producing pathogens difficult to treat; thus, biofilms are an area of concern in human and animal infections.

New strategies to disrupt biofilm formation have shown efficacy *in vitro*, and they usually target the different components that form the extracellular matrix [8, 9, 10]. Mutations in genes encoding extracellular nucleases reduced biofilm formation and were correlated with an increase in daptomycin susceptibility in a murine model, revealing the therapeutic relevance of this strategy [11]. Currently, there is a race to identify substances with antibiofilm activity; which has previously been observed in plant extracts, algae polysaccharides, DNaseI, and proteases [12].

Bovine mastitis is an important disease that affects dairy cattle and can be caused by several microorganisms, mainly bacteria [13]. *Staphylococcus aureus* is one of the most important pathogens that cause clinical and subclinical mastitis [14]. The contagious nature of the bacteria helps them to spread from one animal to the other during milking, which makes preventative management an effective method to control the disease [15].

Antimicrobials are routinely used for the treatment of bovine mastitis although there is a considerable variation in the clinical outcome for cows infected with *S*. *aureus* [16, 17, 18]. Therapeutic failure of antibiotics may be due to the ability of the bacteria to form a well-structured biofilm [1, 19]. Several authors have reported the *in vitro* production of biofilms by bovine mastitis isolates [20, 1, 21], and their various biofilm-producing potentials suggest that biofilms are vital virulence factors, at least for some strains [22].

Snake venoms are a rich source of substances that affect different biological processes, such as neurotransmission, coagulation, and inflammation. Captopril, the first oral angiotensin-converting enzyme (ACE) inhibitor, was isolated from *Bothrops jararaca* and is currently used commercially for the treatment of hypertension [23]. The venom from *Bothrops jararacussu* is rich in metalloproteases, serine proteases, phospholipase A_2_, L-amino acid oxidases, and other components that could be promising new drugs for the treatment of several diseases. In the current study, we report a new biological activity for a snake venom compound. The purified protein was able to interfere with biofilm formation in *Staphylococcus* sp. and was also efficient against pre-formed biofilms. The antibiofilm substance was identified as a C-type lectin. This is the first report describing the antibiofilm activity of a C-type lectin.

## Material and Methods

### Ethics Statement

This study required no approval by the University Ethics Committee on Animal Use (CEUA) based on reasoning that the study dealt with venom milked from snakes of the wild and no laboratory experimentation was conducted with animals. The committee understands that the research was previously released by the Chico Mendes Institute for Biodiversity Conservation (ICMBio), an administrative arm of the Ministry of the Environment (MMA), that has the overall responsibility of supervising all wildlife research in the country (permit no 39126–1). This permit also allowed snake capture and milking that were carried out by a herpetologist from the Department of Animal Biology. The reptile was released into its natural habitat after the procedure. The permission issued by CNPq granted access to genetic heritage (permit no 010548/2013-0). The samples were collected at the Mata da Biologia, a University research land used by students and faculty of the Universidade Federal de Viçosa.

### Bacterial isolates and venom source

The reference strains *Staphylococcus epidermidis* NRS101 (ATCC 35983) and *S*. *aureus* NRS155 (RN 9120) were obtained from NARSA (Network on Antimicrobial Resistance in *Staphylococcus aureus*) and were used as controls in biofilm assays. The other staphylococcal isolates used in this study were kindly provided by Embrapa Dairy Cattle, Juiz de Fora, Minas Gerais. They are strong biofilm producers isolated from mastitic milk collected from animals in herds of southeastern Brazil. Bacteria were grown in Brain Heart Infusion broth (BHI, HiMedia, Mumbai, India) supplemented with 0.25% glucose (BHIg) at 37°C with agitation. All of the isolates used in this study were stored at -80°C in BHI containing 40% glycerol.

*Bothrops jararacussu* venom was manually extracted from snakes collected at Mata da Biologia (20°45'S, 42°52'W), Universidade Federal de Viçosa. The venom was stored frozen at -20°C until use.

### Biofilm production assay

Biofilm formation was assessed in sterile flat-bottomed 96-well polystyrene microtiter plates. The bacterial isolates were inoculated into BHIg at 37°C and grown for 16 h on a rotary shaker at 180 rpm. A cell suspension adjusted to 0.5 McFarland scale was prepared for each bacterial strain and added to the wells containing snake venom fractions or purified C-type lectin. The wells were then filled with BHIg to a final volume of 200 µL. The plates were incubated at 37°C without agitation. Growth was monitored by the optical density (OD) at 600 nm with a microplate reader (VersaMax Molecular Devices, Sunnyvale, USA). After 22 h, the medium was discarded, the wells were gently washed three times with 200 µL of sterile Phosphate buffer saline, pH 7.4 (PBS), and staining was performed with 200 µL of 0.1% crystal violet for 30 min. Each well was re-washed three times with 200 µL of sterile distilled water prior to the addition of 200 µL of 95% ethanol and measurement of the OD_560nm_. Biofilms incubated with PBS only were used as controls. Each isolate was tested in triplicate, and the assay was repeated three times.

For the pre-formed biofilm assay, the biofilm was allowed to grow for 22 h as described above. The compounds were added into the wells and incubated at 37°C for 2 and 3 h. The biofilm disruption was then evaluated.

### Size exclusion and affinity FPLC chromatography

Crude venom was centrifuged at 10,000 x g for 10 min to remove insoluble material. The resulting supernatant was maintained at -20°C for further assays. For size-exclusion chromatography, one aliquot of this sample was suspended in a saline buffer (150 mM NaCl) to a final concentration of 5 mg/mL and applied onto a Superdex Peptide HR 10/30 (GE Healthcare, Buckinghamshire, England) in an FPLC (Fast Protein Liquid Chromatography) System (GE Healthcare). The material was eluted using the same buffer at a constant flow rate of 0.5 mL/min and monitored by absorbance measurements at 280 nm. Fractions (400 µL) were collected and kept at -20°C until the determination of biological activity.

For the C-type lectin purification, the centrifuged venom was suspended in PBS (137 mM NaCl, 2.7 mM KCl, 10 mM Na_2_HPO_4_, 2 mM KH_2_PO_4_) supplemented with 0.5 M NaCl (pH 7.4) to a final concentration of 25 mg/mL and applied onto an agarose-D-galactose column (GE Healthcare) connected to an FPLC system (GE Healthcare). The column was washed with the same buffer at a flow rate of 0.5 mL/min until the absorbance at 280 nm had returned to baseline. The C-type lectin bound to the column was eluted with PBS containing 300 mM D-galactose. The elution profile was monitored by reading the absorbance at 280 nm. The purified lectin was dialyzed against PBS and maintained at -20°C.

All samples were analyzed by SDS-PAGE using 12% polyacrylamide gels (12 x 10 cm, 0.75 mm thickness) under reducing conditions [24].

### Protein in-gel digestion

Bands of interest were extracted from gels, placed in 96-well plates, and washed with water. Tryptic digestion was performed using a MassPrep liquid handling robot (Waters, Milford, USA) according to the manufacturer’s specifications, and the protocol of Shevchenko *et al*. [25] was followed with the modifications suggested by Havlis *et al*. [26]. Briefly, proteins were reduced with 10 mM DTT and alkylated with 55 mM iodoacetamide. Trypsin digestion was performed using 126 nM modified porcine trypsin (sequencing grade, Promega, Madison, WI, USA) at 58°C for 1 h. The digestion products were extracted using 1% formic acid and 2% acetonitrile followed by 1% formic acid and 50% acetonitrile. The recovered extracts were pooled, vacuum centrifuge-dried and subsequently resuspended in 10 µL of 0.1% formic acid. Aliquots (2 µL) of the extracts were then analyzed by mass spectrometry (MS).

### Mass spectrometry

Mass spectrometry analysis was performed at the Proteomics Platform of the Quebec Genomics Center (Centre de recherche du CHU de Québec, CHUL, Québec, QC, Canada) on a TripleTOF 5600 mass spectrometer fitted with a nanospray III ion source (ABSciex, Concord, ON) and coupled to an Agilent 1200 HPLC (High Performance Liquid Chromatography). Samples (2 µL) were injected via the Agilent 1200 autosampler onto a 0.075 mm (internal diameter) self-packed PicoFrit column (New Objective, Woburn, MA, USA) packed with an isopropanol slurry of Jupiter C18 (5 µm; Phenomenex), which served as the stationary phase, using a pressure vessel set at 700 p.s.i. The column length was 15 cm. The samples were run using a 65 min gradient from 5–35% solvent B (solvent A: 0.1% formic acid in water; solvent B: 0.1% formic acid in acetonitrile) at a flow rate of 300 nL/min. Data were acquired using an ion spray voltage of 2.4 kV, curtain gas of 30 PSI, nebulizer gas of 8 PSI, and an interface heater temperature of 125ºC. An information-dependent acquisition (IDA) method was set up with the MS survey range set between 400 amu and 1250 amu (250 msec), followed by dependent MS/MS scans with the mass range set between 100 and 1800 amu (50 msec) of the 20 most intense ions in high sensitivity mode with a 2+ to 5+ charge state. Dynamic exclusion was set for a period of 3 sec and a tolerance of 100 ppm. The Mascot Generic Format (MGF) peak list files were created using Protein Pilot software (version 4.5; ABSciex) utilizing the Paragon and Progroup algorithms [27]. MGF sample files were then analyzed using Mascot (version 2.4.0; Matrix Science, London, UK). Mascot was set up to search the Uniref100-*Homo sapiens* database assuming trypsin digestion. Mascot was searched with a fragment ion mass tolerance of 0.10 Da and a parent ion tolerance of 0.10 Da. The oxidation of methionine was specified as a variable modification, and carbamidomethylation (C) was specified as a fixed modification. Two missed cleavage sites were allowed.

### Criteria for protein identification

Scaffold (version 4.0.1; Proteome Software Inc., Portland, OR, USA) was used to validate the MS/MS-based peptide and protein identifications. The False Discovery Rate for proteins was calculated as the sum of the Exclusive Spectrum Counts of decoy proteins divided by the sum of the Exclusive Spectrum Counts of target proteins, converted to a percentage. The FDR of proteins/peptides was set to 1% or less based on decoy database searching. Protein probabilities were assigned by the Protein Prophet algorithm [28]. Proteins that contained similar peptides and could not be differentiated based on MS/MS analysis alone were grouped to satisfy the principles of parsimony.

### Scanning electron microscopy of biofilms

*S*. *aureus* NRS155 and *S*. *epidermidis* NRS101 biofilms were prepared in polystyrene supports (0.3 x 0.3 x 0.1 cm). The supports were immersed into 96-well microtiter plates containing 100 µL of a 0.5 McFarland bacterial suspension and 100 µL of BHIg. Biofilms were treated using 100 µg/mL of lectin per well. The control consisted of biofilms treated with PBS. The microplates were incubated at 37°C for 22 h without agitation. The medium was discarded, and the wells were gently washed three times with 200 µL of sterile PBS (pH 7.4) and fixed with a 2.5% (w/v) solution of glutaraldehyde in 0.05 M phosphate buffer (pH 7.2) for 2 h. After fixing the cells, the samples were dehydrated using an ethanol series (30%, 50%, 70%, 80%, 95%; 15 min each), followed by three washes in 100% ethanol. The samples were then dried in a critical point dryer (CPD, Bal-tec 030) using liquid CO_2_ and subsequently coated with gold (approximately 15 nm thickness) using a sputter coater (Balzers, FDU 010). The polystyrene supports were examined on a scanning electron microscope (Leo, 1430 VP) at an accelerating voltage of 20 kV [29].

## Results

**Fig 1:**
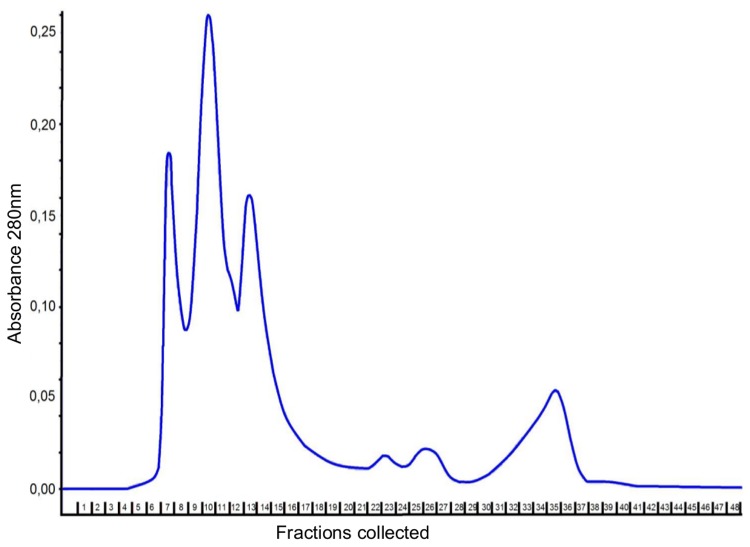
Chromatography of *Bothrops jararacussu* venom. Size-exclusion chromatography of *B*. *jararacussu* venom on a Superdex Peptide HR 10/30 column equilibrated with 150 mM NaCl. The fractions (0.4 mL/tube) were collected at flow a rate of 0.5 mL/min.

**Fig 2:**
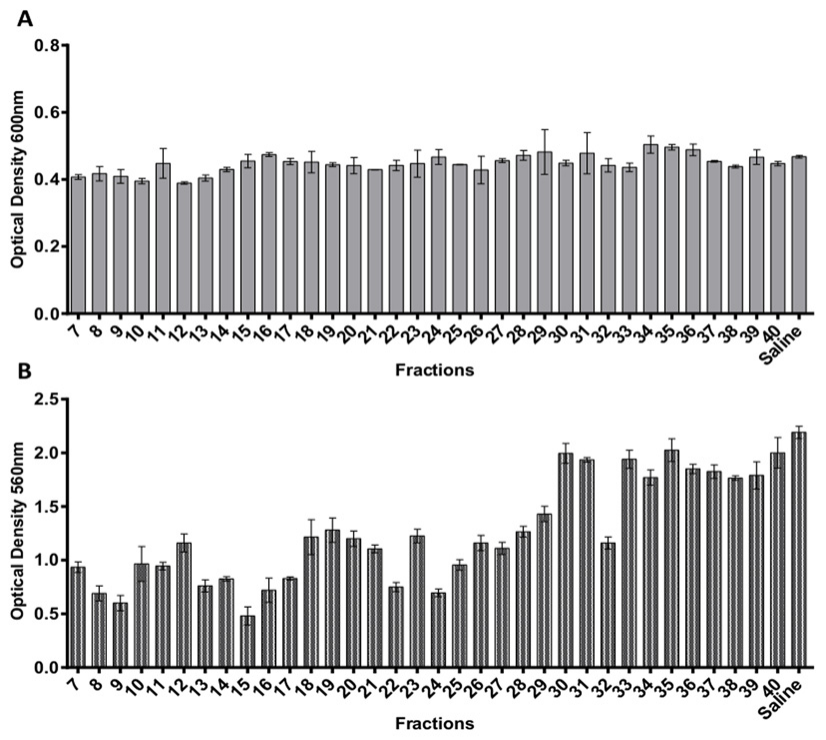
Effect of fractions purified from *Bothrops jararacussu* venom on *Staphylococcus aureus* growth and biofilm biomass. *Staphylococcus aureus* NRS155 was grown in BHI with 0.25% glucose at 37°C for 22 h in contact with the fractions. Bacterial growth was determined by measuring OD_600nm_ (A), and the biofilm biomass was determined by measuring OD_560nm_ (B). The results are the average of three independent experiments ± SD.

**Fig 3:**
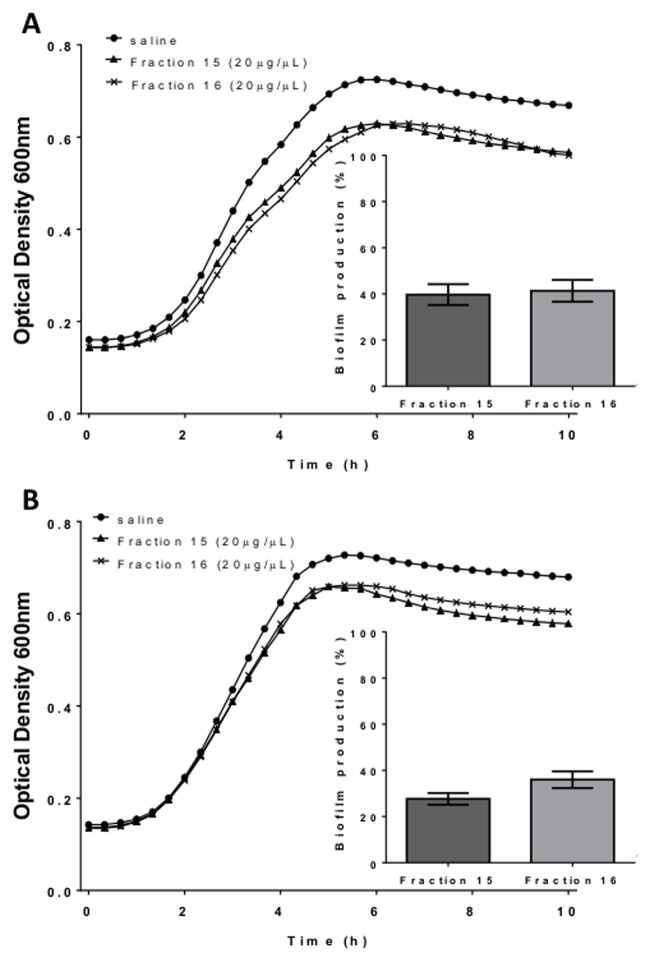
Effect of fractions 15 and 16 on bacterial growth and biofilm production. *Staphylococcus aureus* NRS155 (A) and *S*. *epidermidis* NRS101 (B) were grown at 37ºC in BHIg containing fraction 15 or 16 (or saline for the control). The bacterial growth (OD_600nm_) and biofilm biomass (OD_560nm_) were measured using a multidetection microplate reader. Inlets Biofilm production measured for *S*. *aureus* (A) and *S*. *epidermidis* (B) grown in for 22 h at 37°C in BHIg containing 20 µg/mL of fraction 15 or 16. The percentage of biofilm production was calculated relative to the control (BHIg containing saline instead of fraction 15 or 16) which was set to 100%. Results represent the average of three independent experiments ± SD.

Crude venom of *B*. *jararacussu* was centrifuged and diluted to a concentration of 5 mg/mL. Subsequently, 100 µL aliquots were injected into an FPLC coupled to a Superdex peptide column to separate substances with biological activity against bacteria. Four main peaks were observed, and the first three peaks contained the majority of the proteins (Fig. 1). A total of 48 fractions were recovered and screened for biological activity. The effect of 34 fractions that contained detectable amounts of protein on bacterial cell viability was evaluated by measuring OD_600nm_; however, none of the fractions showed significant growth activity against *S*. *aureus* NRS155 (Fig. 2A). Interestingly, some fractions showed strong antibiofilm activity (Fig. 2B). Several proteins were observed in many of the fractions having antibiofilm activity (e.g., fraction 9, S1 Fig.). However, the strong antibiofilm activity of fractions 15 and 16 was associated to a low diversity and amount of proteins (S1 Fig.). For these reasons, only fractions 15 and 16 were selected for the subsequent assays. A microplate biofilm assay and a growth curve analysis were performed to evaluate whether fractions 15 and 16 were affecting bacterial growth in a way that could promote a reduction in biofilm production (Fig. 3). No significant effect on *S*. *aureus* or *S*. *epidermidis* growth was observed, although the OD_600nm_ value obtained in the stationary phase was slightly lower than that observed in the control group. A comparison of the specific growth rates between cells treated with saline and cells treated with fractions 15 and 16 were performed, and no effect was observed (S1 Table). These results indicate that the fractions may have been inhibiting biofilm formation by other mechanisms that were not greatly affecting bacterial growth.

**Fig 4:**
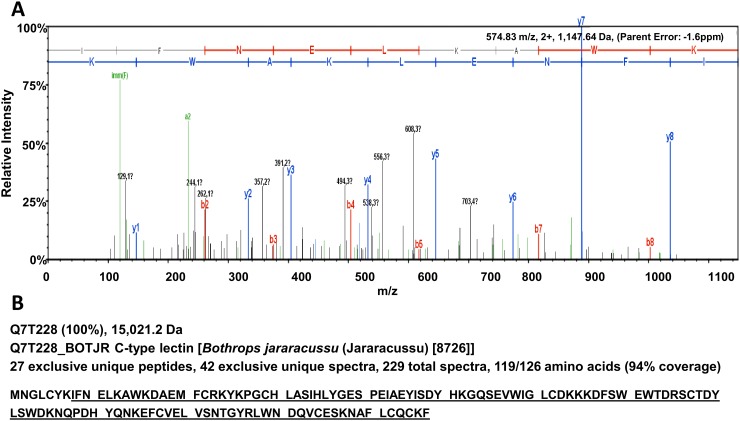
A C-type lectin was identified by TripleTOF 5600 MS. The mass spectrometer was fitted with a nanospray III ion source and coupled to an Agilent 1200 HPLC. A typical spectrum of a single peptide [(K)IFNELKAWK(D), m/z, 574.83] is shown (A). The mass and the amino acid sequence of the C-type lectin are shown and the sequence obtained from spectrometry is underlined (B). This protein was identified as Q7T228 in Uniprot database with coverage of 94%.

Fractions 15 and 16 were analyzed by SDS-PAGE, and a band of approximately 15 kDa was observed (S1 Fig.). This band was extracted from the gel, trypsinized, and analyzed on a TripleTOF 5600 mass spectrometer. A stronger band of the same size (15 kDa) obtained from fraction 15 of whole venom was also sequenced. The ions obtained from the one representative peptide identified by mass spectrometry are shown in Fig. 4 and the complete list of peptides generated by mass spectrometry is presented in S1 Dataset. Considering a minimum of 5 peptides with a threshold of 95%, 27 exclusive unique peptides were found along with 42 exclusive unique spectra. Using these criteria, the protein Q7T228 from Uniprot database was identified as a C-type lectin with 94% coverage (Fig. 4B). The same results were obtained for the two sequenced bands.

**Fig 5:**
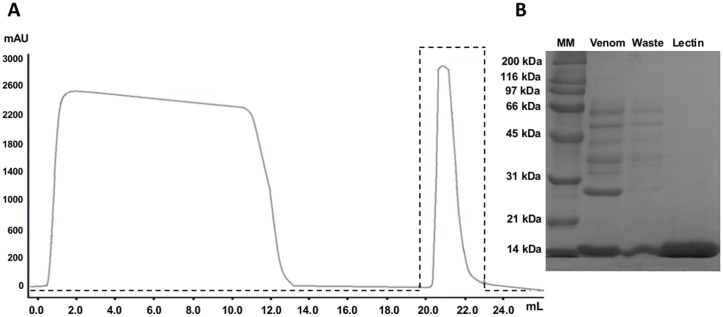
Affinity chromatography of the C-type lectin. The lectin from *Bothrops jararacussu* venom was purified using a D-galactose column (3 cm x 1 cm, I.D.). The adsorbed proteins (lectin) were eluted with PBS (pH 7.4) containing 300 mM galactose (dashed line) (A). The protein concentration was monitored by reading the OD_280nm_ (solid line). SDS-PAGE analysis of the purified lectin from *B*. *jararacussu* venom (B). The gel was loaded with 25 µL of the following samples: Molecular Mass Marker (MM, kDa), crude venom diluted 1:100 in PBS (venom), proteins not bound to D-galactose column (waste), purified protein eluted with 300 mM galactose (lectin).

C-type lectins have galactose-binding properties. For this reason, we performed a single chromatographic step using an agarose-D-galactose affinity column coupled to an FPLC AKTA Purifier UPC10 to isolate the lectin identified by mass spectrometry. The crude venom was centrifuged, diluted in PBS, and applied to the column using a 10 mL loop. Only one peak was obtained when 300 mM galactose was used as the elution buffer (Fig. 5A). A single strong band of ≈15 kDa was found when the eluate was analyzed by SDS-PAGE (Fig. 5B). The waste contained all other proteins initially present in the crude venom.

**Fig 6:**
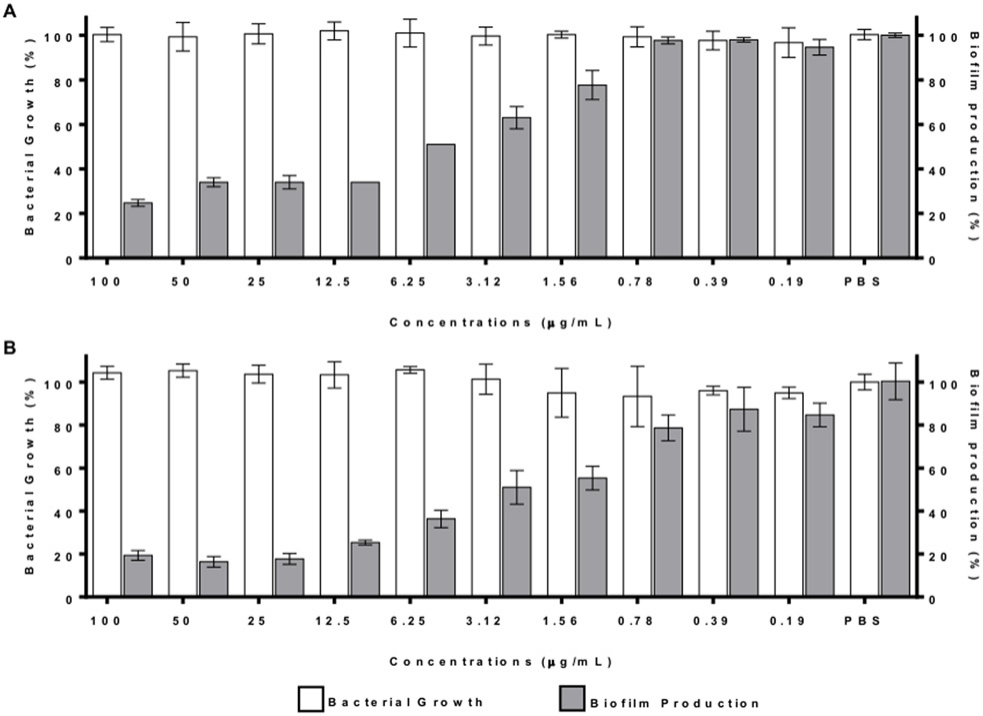
The C-type lectin prevented biofilm formation in a dose-dependent manner. The C-type lectin was incubated at 37°C for 22 h in serial dilutions from 100 µg/mL to 0.19 µg/mL. The bacterial growth (OD_600nm_) and biofilm production (OD_560nm_) of *S*. *aureus* NRS155 (A) and *S*. *epidermidis* NRS101 (B) were monitored. The percentage of bacterial growth and biofilm production was calculated relative to that observed with PBS and expressed in percentage. The results are the average of three independent experiments ± SD.

The antibiofilm activity of the purified lectin was again assayed to evaluate the effect of increased lectin concentrations on *S*. *aureus* (Fig. 6A) and *S*. *epidermidis* (Fig. 6B). A significant dose-dependent effect on the formation of *S*. *aureus* and *S*. *epidermidis* biofilms was observed. The purified lectin did not have an effect on bacterial growth at any concentration tested.

**Fig 7:**
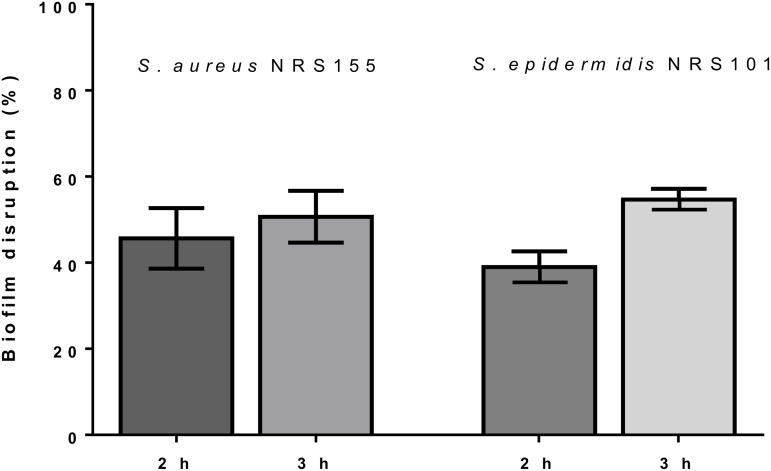
The C-type lectin disrupts pre-formed staphylococcal biofilms. *S*. *aureus* and *S*. *epidermidis* were cultivated in microplates for 22 h at 37°C. The bacteria were washed, and the biofilm cells attached to the microtiter plate wells were incubated with 100 µg/mL C-type lectin for an additional 2 or 3 h. After staining with crystal violet, the OD_560nm_ was measured. The percentage of biofilm disruption was calculated relative to the control (saline solution) which was set to 100%. The percentage of biofilm disruption was calculated relative to that observed with PBS, which served as a control. The values are the means ± SD from three independent experiments.

To evaluate the effect of the lectin on established biofilms, *S*. *aureus* and *S*. *epidermidis* were allowed to grow in BHIg for 22 h (Fig. 7). The cells were then gently washed and incubated with 100 µg/mL lectin for 2 or 3 h. More than 50% of the pre-formed biofilm was disrupted when compared to the control. Also, biofilm disruption was accentuated when the incubation time was extended from 2 h to 3 h.

**Fig 8:**
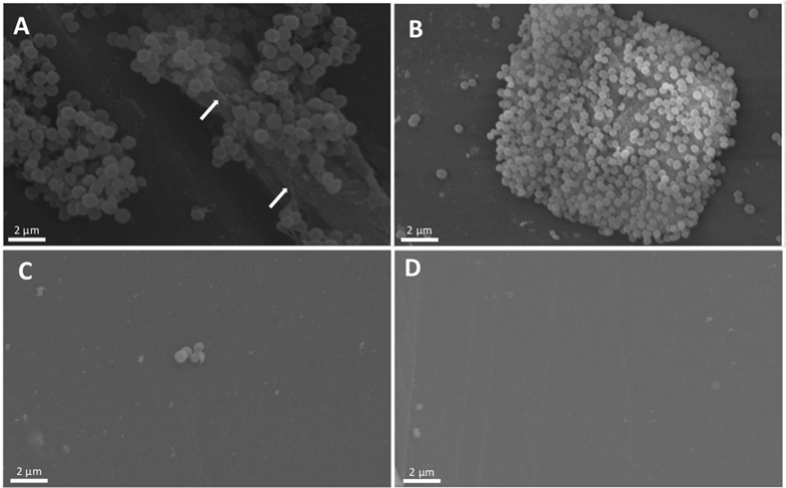
Scanning electron microscopy reveals disruption of staphylococcal biofilms by lectin. *Staphylococcus aureus* NRS 155 (A–C) and *S*. *epidermidis* NRS 101 (B–D) were grown on a polystyrene surface for 22 h at 37°C in the presence (C–D) or absence (A–B) of 100 µg/mL lectin. Arrows indicate staphylococcal extracellular polymeric substance.

Scanning electron microscopy was performed as an alternative means of evaluating the antibiofilm effect of the lectin (Fig. 8). Bacterial cells were treated with 100 µg/mL lectin (Fig. 8C and 8D) or PBS as a control (Fig. 8A and 8B). Cell clusters on polystyrene supports were only seen with the control treatment and were accompanied with an extracellular polymeric substance (EPS) matrix that typically surrounds staphylococcal biofilms (Fig. 8A, arrows). Biofilm production by both bacteria was drastically reduced using the lectin.

**Fig 9:**
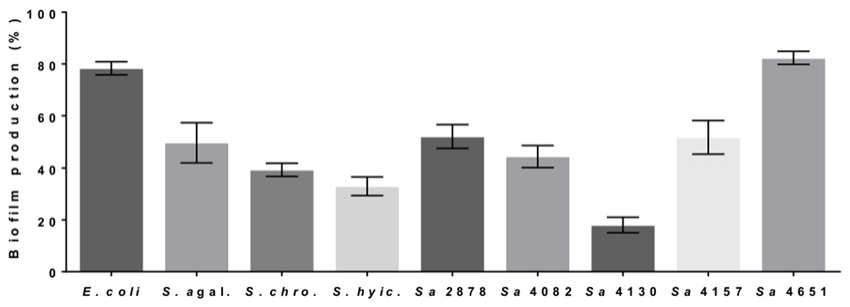
The C-type lectin prevents biofilm production in different bacterial species. The bacteria *E*. *coli* (*Escherichia coli*), *S*. *agal*. (*Streptococcus agalactiae*), *S*. *chro*. (*Staphylococcus chromogenes*), *S*. *hyic*. (*Staphylococcus hyicus*), *Sa* 2878 (*Staphylocccus aureus* 2878), *Sa* 4082 (*S*. *aureus* 4082), *Sa* 4130 (*S*. *aureus* 4130), *Sa* 4157 (*S*. *aureus* 4157) and *Sa* 4651 (*S*. *aureus* 4651) were grown in BHIg containing 50 µg/mL lectin for 22 h at 37°C. Biofilm production was monitored by reading the OD_560nm_ after staining with crystal violet. The percentage of biofilm disruption is shown relative to that observed with PBS, which was used as a control. The values are the means (± SD) of three independent experiments.

We lastly evaluated whether the lectin was also inhibiting biofilm formation in different bacterial species. The lectin showed promising activity against all bacteria studied, including *S*. *hyicus*, *S*. *chromogenes*, *Escherichia coli*, and other bovine mastitis clinical isolates (Fig. 9). Although it showed antibiofilm activity, the lectin did not affect bacterial growth. The growth curves (S2 Fig.) for all tested bacteria were similar, and the comparison of specific growth rates did not reveal any significant differences (S1 Table).

## Discussion

Mastitis is considered the most important disease of dairy cow herds [1]. Although it can be caused by several bacterial species, *S*. *aureus* is recognized as the major cause of bovine mastitis [30, 31]. Infections caused by *S*. *aureus* are difficult to treat, have a low cure rate, and frequently evolve to a chronic state [32]. Different mechanisms have been suggested for pathogen persistence in the udder and their high resistance to antimicrobials and the ability of bovine strains to produce biofilms [33].

A promising strategy for combating staphylococcal infections involves the targeting of bacterial virulence rather than killing the bacteria [34]. Use of such a strategy would make the pathogen less virulent, thus making infections easier to treat [35]. Biofilms are important virulence factors for *S*. *aureus* and other bacteria; thus, they can be considered to be good drug targets. Drugs aimed at extracellular polymeric substances (EPS) are of special interest due to their importance in the arrangement of biofilms formed by diverse microorganisms [36, 37].

Snake venom consists of a pool of different constituents that makes them a rich source of compounds with diverse biological functions [38]. The main contribution of this work was the demonstration of a new biological activity for a protein purified from the venom of *Bothrops jararacussu*, a common species found in the Atlantic Forest of southeastern Brazil. This is also the first time that a lectin has been shown to disrupt bacterial biofilms.

Using size-exclusion chromatography we identified four major peaks with a considerable quantity of proteins. It is well documented that snake venoms are a reservoir of proteins and peptides [39]; however, their antibiofilm activity has not been reported. At the concentration tested, some fractions were able to degrade biofilm without affecting bacterial growth. The fractions that corresponded to the major peaks had more activity than the other fractions. These results may be due to the different protein concentrations that were used in the preliminary screening. At this step, an equal volume of each fraction was used instead of an equal concentration. Thus, fractions that contained greater amounts of active proteins may have shown more pronounced effects. In size-exclusion chromatography, the larger proteins are collected in the earlier tubes, whereas the smaller proteins are collected in the later tubes. However, using this fractionation approach, there is not a complete separation of proteins present in the crude venom. Larger amounts of a specific protein may be present in one tube, while its quantity may be lower in the adjacent tubes. This phenomenon can explain the high antibiofilm activity of fraction number 15 and the reduced activity of the adjacent fractions.

The antibiofilm activity against *S*. *aureus* biofilms encouraged us to evaluate the activity against different microorganisms. Fractions 15 and 16 did not affect bacterial cell viability, and despite the slight difference in the time to reach the stationary phase, the growth rate was similar. The C-type lectin purified by affinity chromatography also very effectively inhibited the biofilms of other pathogens (Fig. 9), although it did not have any effect on the growth of other species. This result suggests that the slight effect on growth caused by fractions 15 and 16 could be due to proteins other than lectin that were present in these fractions.

Biofilm is reduced proportionally to the increase of lectin concentrations. In the presence of 100 µg/mL lectin, the biofilm formation of *S*. *aureus* NRS155 and *S*. *epidermidis* NRS101 was inhibited by 75% and 80%, respectively. Scanning electron microscopy confirmed the antibiofilm effect of lectin, revealing that *S*. *aureus* and *S*. *epidermidis* biofilms were diminished by treatment with 100 µg/mL lectin. The typical biofilm structure, with exopolysaccharides surrounding the cells, was observed only with PBS treatment.

Using a TripleTOF 5600 mass spectrometer coupled to an Agilent 1200 HPLC, we identified a C-type lectin from *Bothrops jararacussu* with 94% coverage. Lectins are proteins with well-characterized properties and many biological activities [39]. These proteins are widely distributed in nature and have been found in animal, fungi and mainly in plants [40]. There are a variety of lectins recognizing different sugars, as fucose, galactose, manose, sialic acid [41] and chitin [42]. The SDS-PAGE excised band that was identified by mass spectrometry was ≈15 kDa, which is the expected molecular weight of *B*. *jararacussu* monomers (15,021 Da). C-type lectins from snake venom are usually described as disulfide-linked dimers containing two homologous polypeptides of approximately 15 kDa that are capable of binding to carbohydrates in a non-covalent manner [43]. Usually, these lectins have a carbohydrate recognition domain (CRD) that binds a sugar moiety and are calcium dependent [44]. This sugar-binding property was used to purify the protein using a D-galactose affinity column and may be part of the mechanism by which C-type lectins disrupt biofilms. Bacterial biofilms contain many different carbohydrates, mainly glucose and galactose, and the compositions vary among species [45]. Thus, considering the variation in the percentage of galactose moieties on biofilms formed by different bacteria, it is reasonable to hypothesize that the antibiofilm activity of lectin can be variable depending on how many galactose residues are present.

The large amount of lectin purified by affinity chromatography suggests that the lectin has a high specificity for galactose; however, this result does not exclude a lower binding affinity for other sugars. The differential affinity of the lectin for different sugars and the variable composition of sugars among bacteria [45] may explain the variable effect on bacterial biofilm disruption. We suggest that C-type lectins may interfere with biofilms via two possible mechanisms. The first mechanism could involve the binding of the CDR domain to carbohydrates present in the biofilm. This binding could disturb biofilm production during bacterial growth. The second mechanism could involve disturbance via a domain that has not been described. Reports describing variable lectin functions have been published [46, 47]. Dos Reis Almeida *et al*. (2010) described the N-acetyl-β-D-glucosaminidase activity of paracoccin, a lectin from *Paracoccidioides brasiliensis* [47]. Similarly, the lectin purified in this work could be a N-acetylglucosamine-degrading protein that acts on the biofilm extracellular matrix. N-acetylglucosamine is ubiquitous [48], which can explain its effect on biofilms formed by different species.

Compared to other lectins described in literature the lectin from *B*. *jararacussu* was very effective in disrupting biofilms at lower concentrations. The lectin isolated from potato promoted a 20% reduction of the biofilm mass of *Pseudomonas aeruginosa* at the concentration of 80 µg/mL [42]. Vasconcelos et al., (2014) described lectins from plants and algae with ability to inhibit bacterial growth and/or biofilm formation in bacteria, including *S*. *aureus* and *S*. *epidermidis*, but none of them were able to reduce biofilm without affecting the bacterial growth [49]. For some of those lectins, the antibiofilm effect was only seen using concentrations as high as 250 µg/mL were assayed. The biological activity of plant lectins on *Streptococcus mutans* was also reported by Islam et al. (2009) [50]. The maximal antibiofilm effect of the mannose/glucose-specific TFA lectin was seen at 100 and 200 µg/mL.

It should be highlighted that aside from the remarkable biofilm disruption, the lectin studied in the work did not affect bacterial growth, which is an important feature of compounds that target bacterial virulence. Compounds not affecting growth would not apply any selective pressure for resistance. Compounds with these features have been considered as alternative strategies for the control of persistent infections, such as those caused by biofilm-forming bacteria [34]. Evaluation of the therapeutic activity of C-type lectin in animal models is underway.

## Conclusions

This study was the first to reveal that compounds in snake venom have effective antibiofilm activity. A C-type lectin was purified by affinity chromatography, and different assays were used to demonstrate a biological activity that has never been described previously for this family of proteins. The C-type lectin had a significant inhibitory effect on several Gram-positive and Gram-negative biofilms but did not have effects on the growth of these species. Because biofilms are important virulence factors that enable bacteria to survive antibiotic therapy and the host immune response, C-type lectin appears to be a promising tool that can be used against biofilm formed by bacteria. Our study provides a new perspective for research on lectins, and studies are under way to evaluate their antibacterial activity *in vivo*.

## Supporting Information

S1 Dataset Peptide fragments observed for the lectin by TripleTOF 5600 mass spectrometer. The properties of each peptide spectrum associated with the lectin were analysed using Scaffold software (version 4.0.7). The string of amino acids in the peptide is prefaced and followed by one residue symbol in parentheses. The symbols in parentheses represent the residue just before and just after the peptide, within the entire protein sequence.
(XLS)

S1 Fig SDS-PAGE analysis of *Bothrops jararacussu* venom fractions obtained from FPLC. The gel was loaded with 25 µL of the following samples: Molecular Mass Marker (MM, kDa), crude venom diluted 1:100 in PBS (V), and fractions 5 to 12 (A) and 13 to 21 (B).
(TIF)

S2 Fig Growth curves of different bacterial species cultivated in the presence of lectin. Each bacteria was grown in BHIg containing PBS or 100 µg/mL lectin for 10 h at 37°C. The bacterial growth (OD_600nm_) was measured using a multidetection microplate reader. The values are the means (± SD) of three independent experiments.
(TIF)

S1 Table Comparison of the specific growth rate µ (h^-1^) of multiple bacterial strains grown under different conditions. (PDF)
