# Supplementary figures and images for: A C-Type Lectin from Bothrops jararacussu Venom Disrupts Staphylococcal Biofilms
Source: PLoS One. 2015 Mar 26;10(3):e0120514. doi: 10.1371/journal.pone.0120514 (PMC4374669; doi:10.1371/journal.pone.0120514)

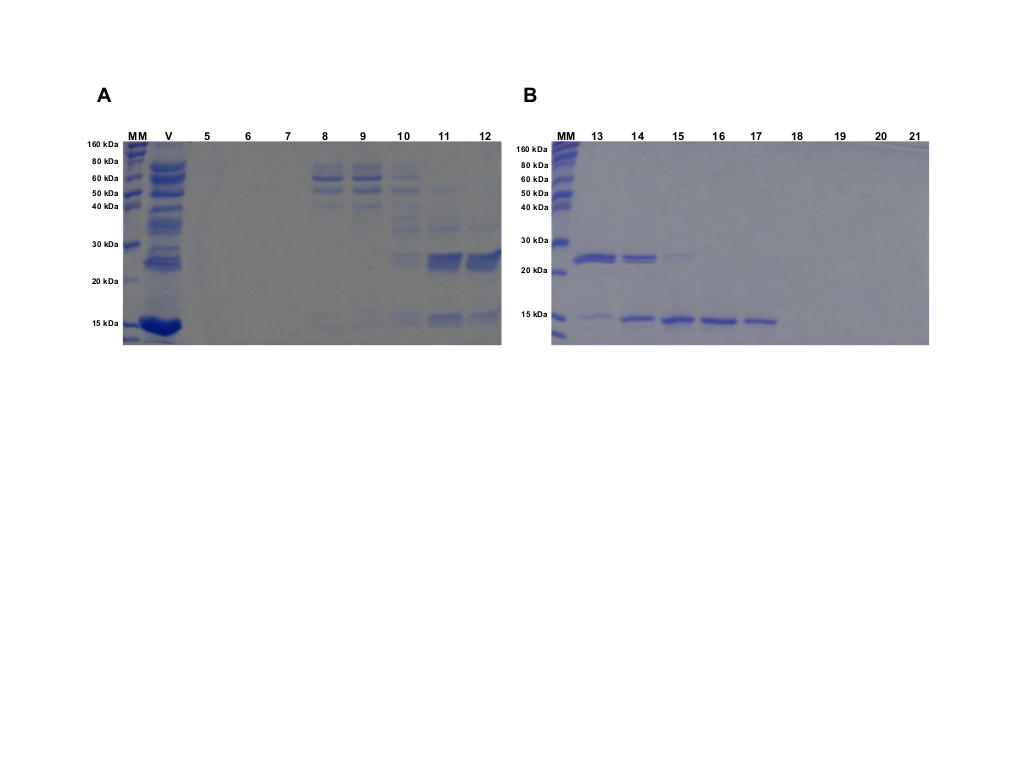

Supplement: S1 Fig — The gel was loaded with 25 µL of the following samples: Molecular Mass Marker (MM, kDa), crude venom diluted 1:100 in PBS (V), and fractions 5 to 12 (A) and 13 to 21 (B). (TIF) [file pone.0120514.s002.tif]

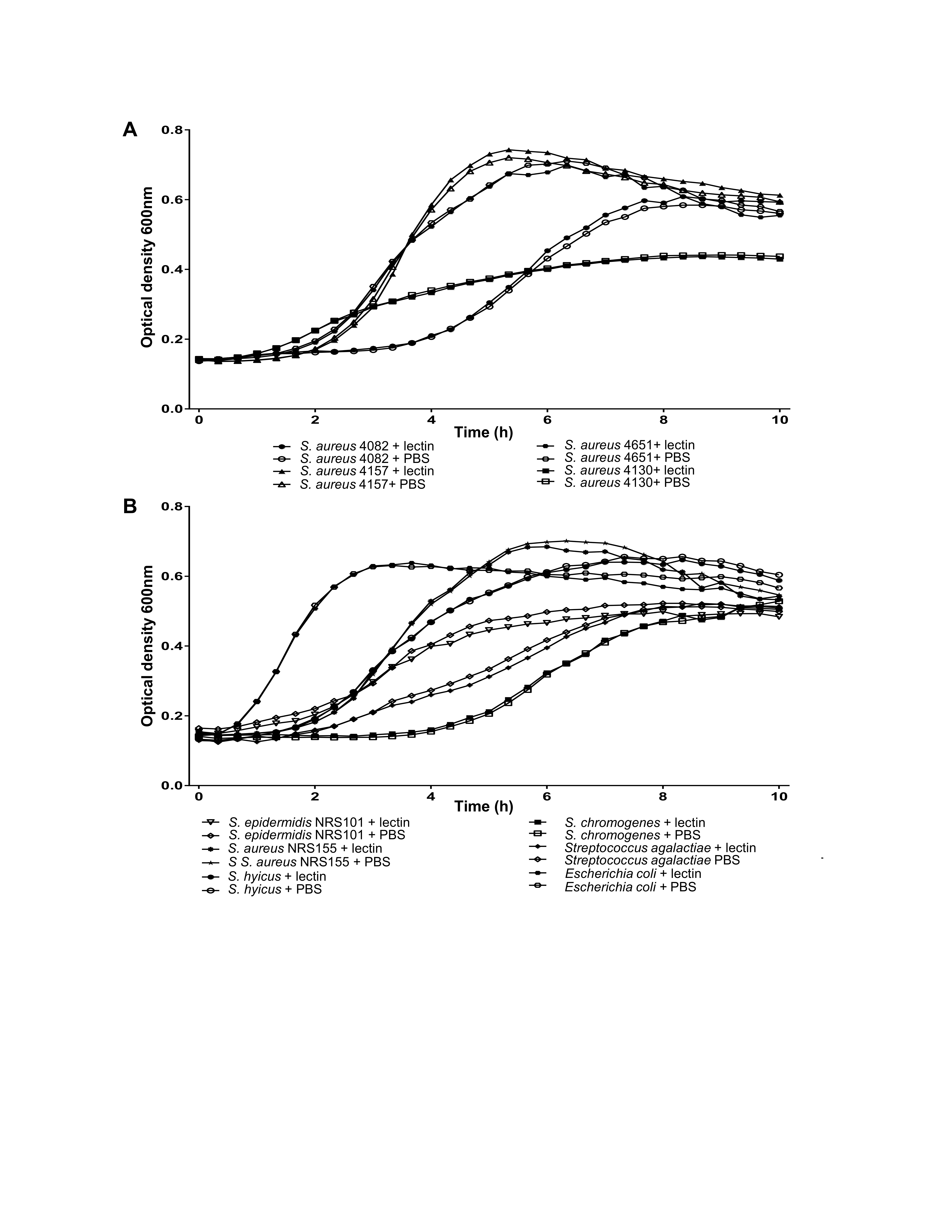

Supplement: S2 Fig — Each bacteria was grown in BHIg containing PBS or 100 µg/mL lectin for 10 h at 37°C. The bacterial growth (OD600nm) was measured using a multidetection microplate reader. The values are the means (± SD) of three independent experiments. (TIF) [file pone.0120514.s003.tif]
