# Supplementary material for: A C-Type Lectin from Bothrops jararacussu Venom Disrupts Staphylococcal Biofilms
Source: PLoS One. 2015 Mar 26;10(3):e0120514. doi: 10.1371/journal.pone.0120514 (PMC4374669; doi:10.1371/journal.pone.0120514)
Supplement: S1 Table — (PDF) [file pone.0120514.s004.pdf]

Table S1. Comparison of the specific growth rate μ (h^{-1}) of multiple bacterial strains grown under different conditions.

Organism	Conditions				
	**Saline**	**Fraction 15**	**Fraction 16**	**PBS**	**Lectin**
Staphylococcus epidermidis NRS101	0.1898	0.1603	0.16989	0.1251	0.1204
Staphylococcus aureus NRS155	0.1484	0.1248	0.1217	0.1578	0.1544
Staphylococcus aureus 4082				0.1125	0.1186
Staphylococcus aureus 4157				0.1860	0.1977
Staphylococcus aureus 4130				0.0612	0.0593
Staphylococcus aureus 4651				0.1524	0.1523
Staphylococcus chromogenes				0.0878	0.0883
Staphylococcus hyicus				0.1229	0.1209
Staphylococcus agalactiae				0.0448	0.0454
Escherrichia coli				0.1728	0.1722
